# Cues Disseminated by Professional Associations That Represent 5 Health Care Professions Across 5 Nations: Lexical Analysis of Tweets

**DOI:** 10.2196/42927

**Published:** 2023-03-15

**Authors:** Ann Dadich, Rebecca Wells, Sharon J Williams, Nazim Taskin, Mustafa Coskun, Corinne Grenier, Frederic Ponsignon, Shane Scahill, Stephanie Best

**Affiliations:** 1 School of Business Western Sydney University Parramatta Australia; 2 Department of Management, Policy and Community Health, University of Texas Texas, TX United States; 3 School of Health & Social Care, Swansea University Swansea United Kingdom; 4 Department of Management Information Systems, Boğaziçi University Istanbul Turkey; 5 Kedge Business School Talence France; 6 Faculty of Medical and Health Sciences, University of Auckland Auckland New Zealand; 7 Peter MacCallum Cancer Centre Melbourne Australia

**Keywords:** professional associations, social media, professional identity, collaboration, knowledge translation

## Abstract

**Background:**

Collaboration across health care professions is critical in efficiently and effectively managing complex and chronic health conditions, yet interprofessional care does not happen automatically. Professional associations have a key role in setting a profession’s agenda, maintaining professional identity, and establishing priorities. The associations’ external communication is commonly undertaken through social media platforms, such as Twitter. Despite the valuable insights potentially available into professional associations through such communication, to date, their messaging has not been examined.

**Objective:**

This study aimed to identify the cues disseminated by professional associations that represent 5 health care professions spanning 5 nations.

**Methods:**

Using a back-iterative application programming interface methodology, public tweets were sourced from professional associations that represent 5 health care professions that have key roles in community-based health care: general practice, nursing, pharmacy, physiotherapy, and social work. Furthermore, the professional associations spanned Australia, Canada, New Zealand, the United Kingdom, and the United States. A lexical analysis was conducted of the tweets using Leximancer (Leximancer Pty Ltd) to clarify relationships within the discourse.

**Results:**

After completing a lexical analysis of 50,638 tweets, 7 key findings were identified. First, the discourse was largely devoid of references to interprofessional care. Second, there was no explicit discourse pertaining to physiotherapists. Third, although all the professions represented in this study support patients, discourse pertaining to general practitioners was most likely to be connected with that pertaining to *patients*. Fourth, tweets pertaining to pharmacists were most likely to be connected with discourse pertaining to *latest* and *research*. Fifth, tweets about social workers were unlikely to be connected with discourse pertaining to *health* or *care*. Sixth, notwithstanding a few exceptions, the findings across the different nations were generally similar, suggesting their generality. Seventh and last, tweets pertaining to physiotherapists were most likely to refer to discourse pertaining to *profession*.

**Conclusions:**

The findings indicate that health care professional associations do not use Twitter to disseminate cues that reinforce the importance of interprofessional care. Instead, they largely use this platform to emphasize what they individually deem to be important and advance the interests of their respective professions. Therefore, there is considerable opportunity for professional associations to assert how the profession they represent complements other health care professions and how the professionals they represent can enact interprofessional care for the benefit of patients and carers.

## Introduction

### Background

Systemic challenges in addressing the COVID-19 pandemic have drawn increased attention to the complexities of health care delivery [1]. These include the ways in which different health care professionals work together [2,3]; for instance, although interprofessional care is deemed best practice [4], during the COVID-19 pandemic, some health care professionals “experienced ambiguous professional identities and role confusion in interprofessional teams” [5].

Even before the COVID-19 pandemic, the increasing prevalence of chronic disease worldwide underscored the need for different health care professionals to work together—to be in each other’s sphere of practice [6-9]. This is because the complementary knowledge and skills of different health care professionals are critical to the care of patients with complex and chronic conditions, such as congestive heart failure, chronic obstructive pulmonary disease, heart disease, and cognitive disorders [10,11]. As the post-COVID-19 condition results in additional chronic disease [12], the need for complementary knowledge and skills will continue to rise.

As important as interprofessional care is for the increasing numbers of patients, such collaboration does not occur automatically [13]. Different professions typically have discrete roles in patient care, are based in disparate departments or organizations, have different payers and regulations, and record information into separate data systems. Professions often compete for domains within patient care, such as assessment and treatment planning [14]. Furthermore, different organizations often have diverse priorities—although they might all espouse the importance of patient well-being, they might pursue this aspiration by reducing patient costs, reducing societal costs, or applying a blend of these at different times [15]. Interprofessional teams also typically include multiple professional cultures, habits, and identities that can hinder collaboration [16]. Therefore, interprofessional care requires both systemic and personal commitment [17]. However, it has been challenging to create the ethos to support such care [18].

Contexts in which different health care professionals work together best are characterized by “a shared purpose, critical reflection, innovation, and leadership” [19-21]. However, there is a limited understanding of what fosters such contexts [22,23]. Health care professionals tend to use their respective disciplines, in addition to local cues from organizational leaders and peers, to guide their practice. The salience of professional identity in health care raises the question of what cues the professional associations provide to their members regarding their respective roles in patient care. In addition, the globally increasing need for interprofessional care does not mean that professional norms are internationally consistent. Thus, there is also a question of how the messaging of professional associations varies across nations.

This study identifies salient themes across professional associations in 5 high-income nations, all of which espouse interprofessional care: Australia, Canada, New Zealand, the United Kingdom, and the United States [24-26]. Specifically, this study presents a lexical analysis of the tweets posted by professional associations in these nations that represent 5 professions with key roles in community-based health care: general practice, nursing, pharmacy, physiotherapy, and social work. Enhanced cooperation across health care roles entails new ways of thinking, which raises the question of how to foster such mindsets [27]. This inquiry reflects the ongoing influence of professional associations on professionals’ frames of reference for their work. Given the popularity of Twitter for professional communication [28-31], tweets were examined to reveal what professional associations deem to be salient.

Before presenting the study, this paper commences with a social identity perspective on health professional communication, given that professions are closely related to social identity [32-34]. Next, the paper describes the method used to examine professional associations’ use of Twitter as well as the associated results. The paper concludes with key findings and the associated implications for scholars and professional associations.

### The Social Identity of Professions

Social identity theory asserts that individuals define themselves largely in comparison with proximate others. Specifically, individuals tend to see themselves as belonging to *in-groups* that they view more favorably than they view members of *out-groups* [35,36]. The assumed motivation behind such biases is a desire to maintain a positive self-image because individual esteem rises along with group esteem [37].

The nature of these comparisons varies according to which social categories are prominent in any given context. In health care, one such categorization is that of professions, which are visible and continually re-emphasized through distinct roles, status, and identity. Professional identity is understood to be “an organized group’s norms, values, and behavioral knowledge that situate an individual into group membership” [38]; in other words, it is “the relationship between the collective level of the profession and the individual level of the professional” [36]. The tertiary education of health care professionals not only qualifies them to practice in their field but also imparts a sense of identity with their profession, along with their responsibilities [39]. However, in addition to tertiary education, professional identity is influenced by myriad factors, including gender, cultural background, and professional ethos, as well as working experience [40-43]. As such, the development of professional identity is iterative [36], and it can become more salient via interactions with different professions; for instance, nurses become more aware of their nursing identity when they interact with physicians or social workers than when they work solely with other nurses [36].

In interprofessional contexts, questions such as *Who am I?* are likely to involve professional identity (eg, *nurse*), and questions such as *What should I do?* are likely to involve professional referent groups (eg, other nurses). This might partly account for health care professionals’ “tribal behaviors”—even on social media—whereby they “communicate within their own profession and within a clinical specialty” [44]. After formal training, the most salient referent group might be a professional association [36,45].

### How Membership Associations Shape Professional Identity

Many health care professionals and those studying to join the profession are members of a professional association, that is, “an organisation with individual members practicing a profession or occupation in which the organisation maintains an oversight of the knowledge, skills, conduct and practice of that profession or occupation” [46]. Indeed, the formation of an association is a key stage in the professionalization process [47]. Member benefits typically include contemporary information on factors that can affect workplace practices, such as trends in patient needs, regulatory changes, and any given profession’s scope of practice. Professional associations also normally provide ongoing member education, including that needed to maintain registration and licenses, and often publish periodicals to advance practice. In addition, many hold annual meetings and conferences at which members establish and nurture relationships with colleagues and learn about developments in their field. Through these mechanisms, professional associations can shape members’ references for interprofessional practice [48].

One of the primary ways that professional associations serve their members is through ongoing communication, increasingly through social media. This includes both factual updates and “a range of professional scripts” to recreate meaning in dynamic contexts [36]. The substance and tone of such interpretive cues are part of the social context of interprofessional care. Professional associations, such as the Royal College of Nursing in the United Kingdom, have reported using social media accounts to share important information with, and cascade it to, members and other stakeholders [49]. The organization recognized social media as fundamental to campaigns to raise awareness of, and support for, member priorities. Members of the Royal College of Nursing were encouraged to incorporate activity on the web into their continuing professional development hours for revalidation and expand their networks with other nurses and professionals. Similarly, as the American Nurses Association’s website noted, “Social media is now a daily part of all our lives. It can not only be entertaining and informative, but it also has the potential to help your career as a nurse and the nursing profession in general” [50]. Even as the COVID-19 pandemic ends (or continues), the recent global shift to web-based communication is likely to increase the prominence of social media.

Among the various social media platforms, Twitter plays a particularly prominent role for health care professionals [51]. Twitter allows users to post short text messages (of up to 280 characters) to others directly (for instance, by replying to a tweet) or indirectly (by mentioning another user or including a hashtag that another user might search for). Other Twitter users can forward (or retweet) messages or might be encouraged to visit a website by accessing embedded hyperlinks. Such flexibility and convenience have attracted increasing users, recently estimated at 350 million, all across the world [52]. This is notable because research suggests that tweets correlate with public opinion [53].

Because of its accessibility, reach, and lack of fees, Twitter has advantages for nonprofit organizations, such as professional associations, which are usually constrained by a limited marketing budget [54]. Twitter, along with other forms of social media, offers nonprofit organizations an opportunity to strengthen support for the organization and its brand [55].

Research offers glimpses into the role of social media among different individual professions [56]; for example, pharmacists are reported to be high users of social media to expand their networks, rather than for education or professional development [57]. A recent study examined social media content to explore public perceptions of interprofessional teams. The authors found that social media can be used to demonstrate the breadth of interprofessional care and highlight the value of less visible professions [58]. However, there is limited research on professional associations’ use of social media, particularly comparative international research.

The global reach of social media conveys an impression of uniformity across the world. However, there has been little empirical examination of where health care dynamics are similar or divergent. Even among high-income nations with commonalities in national origins, there are striking differences in health care [59]. For instance, the United States differs from other high-income nations, such as Australia, in lacking a national health care system. Similarly, although the health systems in the United Kingdom and New Zealand emphasize “*preventive care, safe care, coordinated care,* and *engagement and patient preferences*,” some have suggested that the Canadian health system does not do so [60] (in the quoted text, the italicization reflects the original presentation). Such structural differences reflect distinct national histories and cultures, often characterized in the prominent framework developed by Hofstede [61] in terms of individualism versus collectivism, power distance, uncertainty avoidance, *masculinity* (with an emphasis on power) versus *femininity* (with an emphasis on nurturing), and long-term versus short-term orientation. As researchers have noted, such differences in national culture can shape the nature of professional collaboration [62]; for instance, the absence of a national health care system in the United States partly originates in relative emphases on individualism and the short-term. In turn, such cultural attributes might affect how professionals interpret their respective roles [62].

Given the importance of interprofessional care and the attendant challenges, the aim of this study was to clarify a potentially important normative influence, namely the cues that professional associations disseminate. This was achieved via a lexical analysis of tweets across 5 professions and 5 nations.

## Methods

### Sample

To clarify how professional associations used social media, a sample of tweets was sourced from 25 professional associations across Australia, Canada, New Zealand, the United Kingdom, and the United States (Table 1). These nations were selected because they represent high-income English-speaking nations [63]. The main professional association in each nation that represented each of the following professions was identified: general practice, nursing, pharmacy, physiotherapy, and social work. These disciplines were selected because they commonly work together in community-based health care, that is, “integrated, accessible health care services by clinicians who are accountable for addressing a large majority of personal health care needs, developing a sustained partnership with patients, and practicing in the context of family and community” [64]. Although the 5 aforementioned nations sometimes had more than 1 professional association to represent a profession (eg, the Australian College of Nursing and the Australian College of Midwives in Australia, as well as the American Association of Family Physicians and the American Medical Association in the United States), only the professional association specific to 1 of the 5 aforementioned professions was included in this study.

**Table 1:** Professional associations (n=25).

Nation	General practice	Nursing	Pharmacy	Physiotherapy	Social work
Australia	Royal Australian College of General Practitioners	Australian College of Nursing	Australian Pharmaceutical Society	Australian Physiotherapy Association	Australian Association of Social Workers
Canada	College of Family Physicians of Canada	Canadian Nurses Association	Canadian Pharmacists Association	Canadian Physiotherapy Association	Canadian Association of Social Work
New Zealand	Royal New Zealand College of General Practitioners	Nurses Society of New Zealand	Pharmaceutical Society of New Zealand	Physiotherapy New Zealand	Aotearoa New Zealand Association of Social Workers
United Kingdom	Royal College of General Practitioners	Royal College of Nursing	Royal Pharmaceutical Society	Chartered Society of Physiotherapy	British Association of Social Workers
United States	American Association of Family Physicians	American Nurses Association	American Pharmacists Association	American Physical Therapy Association	National Association of Social Workers

An iterative approach was used to collect the tweets because the Twitter developer application programming interface only allowed 100 of the most recent tweets to be collected. Using a back-iterative application programming interface methodology, the maximum number of public (rather than private) tweets was retrieved from the 25 accounts. This culminated in a collection of 52,440 tweets, posted from June 12, 2018, to October 20, 2020 (approximately 28 months). To optimize comparability, non-English tweets were removed; these included French tweets from Canada (1801/52,440, 3.43%) and a Spanish tweet from Australia. Thus, of the 52,440 tweets collected, 50,638 (96.56%) were used for the lexical analysis.

To optimize the likelihood of a systematic approach to the lexical analysis [65], we used Leximancer data mining software (Leximancer Pty Ltd), which uses Bayesian reasoning to detect key concepts and reveal their relationships [66]. Using algorithms, Leximancer “transform[s] lexical co-occurrence information from natural language into semantic patterns in an unsupervised manner” [67]. The software identifies frequently occurring and co-occurring words and amalgamates these to form and visually map concepts that reflect themes within the text [68]. The maps convey 3 types of information: “the main concepts in the text and their relative importance; the strengths of links between concepts (how often they co-occur); and similarities in contexts where links occur” [69]. Concepts represent “collections of words that generally travel together throughout the text” [70]. The components of these concepts are ordered within a thesaurus, comprising relevant words and weightings to indicate relative importance. Within the map, connections among concepts that are most probable are represented by a spanning tree of gray lines or branches, specifically, “The spanning tree...shows the most-likely connections between concepts (like a road map of highways), but there are other (less-strong) connections between concepts (like backstreets)” [71]. Clusters of concepts within a map—known as themes—suggest contextual similarity [72]. Themes are color coded to signify those that are (and are not) important, whereby the “most important theme appears in red, and the next hottest in orange, and so on according to the colour wheel” [70].

In addition to the novelty of a lexical analysis using Leximancer, compared with other approaches to analyze qualitative data (eg, thematic analyses [73-76]), there are 2 advantages to using this software. First, Leximancer can manage a large corpus of qualitative data, akin to those sourced from social media platforms. Second, compared with other forms of computer-assisted qualitative data analysis software (eg, NVivo [QSR International]), the researcher is at a greater arm’s length of the analysis when using Leximancer—this can reduce researcher bias and increase reliability because the researcher is encouraged to “discover” [77,78] how discourse travels together [79]. These reasons might partly explain growing interest in lexical analyses using Leximancer [80-84].

Leximancer was used in 3 steps. First, we uploaded an Excel (Microsoft Corp) file of all tweets into Leximancer and then used the “discovery mode” to “see what concepts were automatically generated by Leximancer without intervention” [85]. Although Leximancer automatically removes words with low semantic content (eg, “the” and “is”), we manually reviewed the concepts to identify those that should be merged because they were semantically and conceptually similar [86]. Those that we merged included *COVID* and *Covid19*, *GP* (a reference to *general practitioner*) and *gps*, *Join* and *join*, *NASW* (a reference to the National Association of Social Workers in the United States) and *nasw*, and *patient* and *patients*. Furthermore, we removed the concepts *re* and *ve* because they tended to be separated from words such as “you” within a tweet; as such, they offered no conceptual value. We reclustered the map several times to ensure that the concepts were stable. Next, we analyzed the discovery mode concept map by considering the key clusters of concepts represented within the tweets, particularly those relevant to the 5 professions of interest, as described in the *Results* section.

Second, we used Leximancer to examine the comparative importance of the concepts, as denoted by relevance percentage. A relevance percentage represents “the percentage frequency of text segments which are coded with that concept, relative to the frequency of the most frequent concept in the list...This measure is an indicator of the relative strength of a concept’s frequency of occurrence” [87]. In addition to examining the concept with the greatest relevance percentage (100%), we considered the relevance percentages between the concept with the greatest relevance percentage and the concepts that denoted the 5 professions of interest, given the focus of this study.

Third and last, for comparative value, we associated each tweet with 2 tags. Tagging helps to compare the conceptual content of different data [71]. To determine whether (and how) geography influenced the professional associations’ contributions to the Twittersphere, we tagged each tweet according to the nation where the professional associations were located, namely Australia, Canada, New Zealand, the United Kingdom, and the United States. Furthermore, to determine the influence of the profession, each tweet was tagged according to the profession represented by each professional association, namely general practice, nursing, pharmacy, physiotherapy, and social work. Subsequently, we set the *concept generality* to 12 concepts to ensure that those that were mined were not too broad; informed by previous research [88], we enabled the *Learn from Tags* function to ensure that Leximancer searched for concepts associated with each tag independently, disabled the number of concepts to discover “to allow the software to identify the number of relevant concepts,” and selected the *Themed Discovery* option of *Concepts in Each* to “discover concepts that distinguish...categories from one another” [70]. For succinctness, we focused on the concept that was most likely to share the most content with each tag concept, as indicated by the likelihood percentage. Specifically, we analyzed the likelihood of shared content, as described in the *Results* section. As calculated by Leximancer, the likelihood percentage denotes the proportion of text segments that is shared by a tag concept and another concept, thus providing both directions of conditional probability [87].

### Ethical Considerations

This study solely involved an analysis of secondary data, namely tweets in the public domain posted by the official Twitter handles of professional associations. For this reason, the approval of a human research ethics committee was not sought.

## Results

### Key Themes

**Figure 1:**
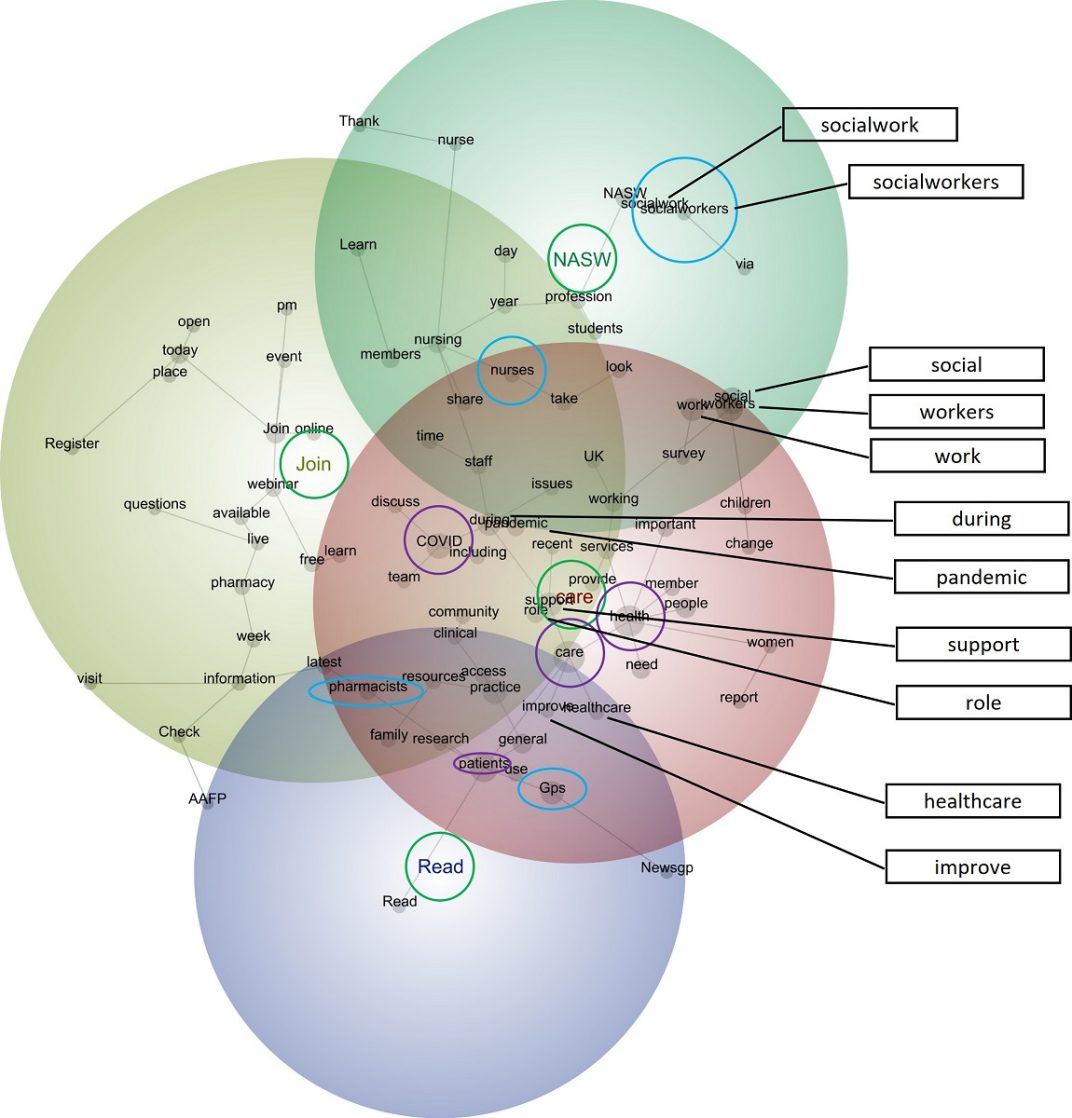
Discovery mode concept map (visible concepts: 100%, theme size: 75%). Words circled in green denote themes, words circled in blue denote professions, and words circled in purple denote the 4 most frequent concepts. In terms of themes, red denotes the most important theme, followed by yellow, green, and purple. AAFP: American Association of Family Physicians; GP: general practitioner; NASW: National Association of Social Workers.

The discovery mode concept map revealed 4 themes: *care*, *Join*, *NASW*, and *Read* (Figure 1). These highlight the key clusters of concepts represented within the text. Theme position illustrates the relationships among the themes, as calculated by Leximancer using Bayesian reasoning [66]. Consider the prominence of *care*, which appears in red and overlaps with the 3 other less prominent themes, particularly *Join*. This suggests that when the tweets referred to *care* (and the concepts therein), they tended to refer to *Join* (and the concepts therein):

In a shifting landscape, tools and ways of working must evolve to meet the needs of patients, GPs and clinical teams. Join the #RCGPConnex networking event and meet like-minded people committed to finding new ways to improve primary care

Join...[these MDs] for Managing The COVID-19 Crisis: Maternal Care During the COVID-19 Pandemic and gain knowledge on how to adjust your practices to maintain the level of care for essential woman s health services. Tune in live on Friday at 7 p.m. CT

The absence of a concept (a gray circle accompanied by a word) that denoted physiotherapy is curious. Although the discovery mode concept map includes the concepts *GPs*, *nurses*, *pharmacists*, and *socialworkers*, it is devoid of a concept pertaining to physiotherapists. This suggests that discourse pertaining to physiotherapists—regardless of who tweeted it—was not prominent within the data set compared with that pertaining to their professional counterparts:

When is it okay for GPs not to tell parents of their child’s medical treatment?

High caseloads risk driving away #socialworkers

Of the 4 other professions, the concept *GPs* was most likely to be connected with the concept *patients*—this is indicated by the gray line between them. This suggests that discourse pertaining to this profession travelled with that pertaining to patients; conversely, discourse pertaining to the other professions was unlikely to travel with that pertaining to patients—the people they work with and support:

GPs have a really important role in ‘selling’ weight loss the right way to their patients and helping them on their journey. [One] Dietitian...gives GPs 10 tips on how you can sow the seed for how they can have an exciting change in their lifestyle

Compared with the other 4 professions, the concept *nurses* was most likely to be connected with the concept *take*, as indicated by the gray line between them. This latter concept included active language, encouraging tweet recipients to ignite action. Conversely, discourse pertaining to the other 4 professions was unlikely to travel with such active language:

Since the changes to student funding, nearly 900 fewer students were due to start on nursing degree courses this year in England. The Government must fix this. Email your MP and ask them to take action to #FundOurFuture nurses

Unlike the other 4 professions, the concept *pharmacists* was most likely to be connected with the concepts *latest* and *research*. As such, when the tweets spoke of this profession, they were likely to be framed as pertaining to contemporary matters, including studies to advance knowledge. Moreover, when the tweets spoke of the other professions, they were unlikely to speak of the concepts *latest* and *research*:

Drug Discovery and Development is the latest e-book available to members via #RPSLibrary . Highlighting scientific advances, future trends and the role of pharmacists in research, it s a must read

Finally, the concept *socialworkers* was most likely to be connected with the concepts pertaining to this profession, namely *socialwork* and *NASW*. Curiously, it was also connected with the concept *via*, which served to direct tweet recipients to particular Twitter handles or websites. This suggests that discourse pertaining to the other professions was unlikely to be connected with this concept and, as such, relatively unlikely to direct recipients to other sources of information:

MI Social Workers Call for Change Ahead of Midterm Elections... #nasw… #ElectionDay

The concept *health* was the most frequent, as denoted by its relevance percentage of 100% (Table 2). This was followed by the concepts *care* (92%), *patients* (87%), and *COVID* (71%), among others. As “indicator[s] of the relative strength of a concept’s frequency of occurrence” [87], this means that the discourse pertaining to these subsequent concepts was highly relevant to that pertaining to *health*, compared with that pertaining to the other concepts:

“There are elements of our health care system that are failing our most vulnerable.”...MACN, Chair of the ACN Advanced Practice COI. #ACNinParliament

#Pharmacists can help steer teen patients with #diabetes toward long-term good health

Today, we d like to highlight how physiotherapy can help in the recovery from COVID-19. Get all the information here...and find out why physiotherapy is key to your overall health

**Table 2:** Ten most frequent concepts.

Concept		Count (relevance: %)	
health		5186 (100)	
care		4748 (92)	
patients		4490 (87)	
COVID		3679 (71)	
workers		3458 (67)	
social		3395 (65)	
GPs^a^		3241 (62)	
support		3166 (61)	
practice		3040 (59)	
work		3037 (59)	
**health**			
	GPs		460 (14)
	nurses		218 (14)
	pharmacists		253 (14)
	socialworkers		73 (7)
**care**			
	GPs	600 (19)	
	nurses	258 (17)	
	pharmacists	259 (14)	
	socialworkers	29 (3)	

^a^GP: general practitioner.

### Relationships Among Concepts

In the context of a study on professional associations that represent health care professions, the frequency of, and relationships among, the concepts *health*, *care*, *patients*, and *COVID* (as calculated using Bayesian reasoning [66]) are perhaps unsurprising. However, the relationships between these salient concepts and the concepts that denote the professions represented by the associations are noteworthy; for instance, compared with the frequent concept *health*, the concepts *GPs*, *nurses*, and *pharmacists* each had a relevance percentage of 14%, whereas the concept *socialworkers* had a relevance percentage of 7% (Table 2). Similarly, compared with the second most frequent concept *care*, the concepts *GPs* (19%), *nurses* (17%), and *pharmacists* (14%) each had a relevance percentage that was greater than that of *socialworkers* (3%). These findings suggest that discourse pertaining to *socialworkers* was unrelated to that pertaining to the salient concepts *health* or *care* in comparison with the other 4 professions. This suggests that discourse pertaining to the profession of social work was somewhat separate or distinct from that pertaining to general practice, nursing, and pharmacy.

**Figure 2:**
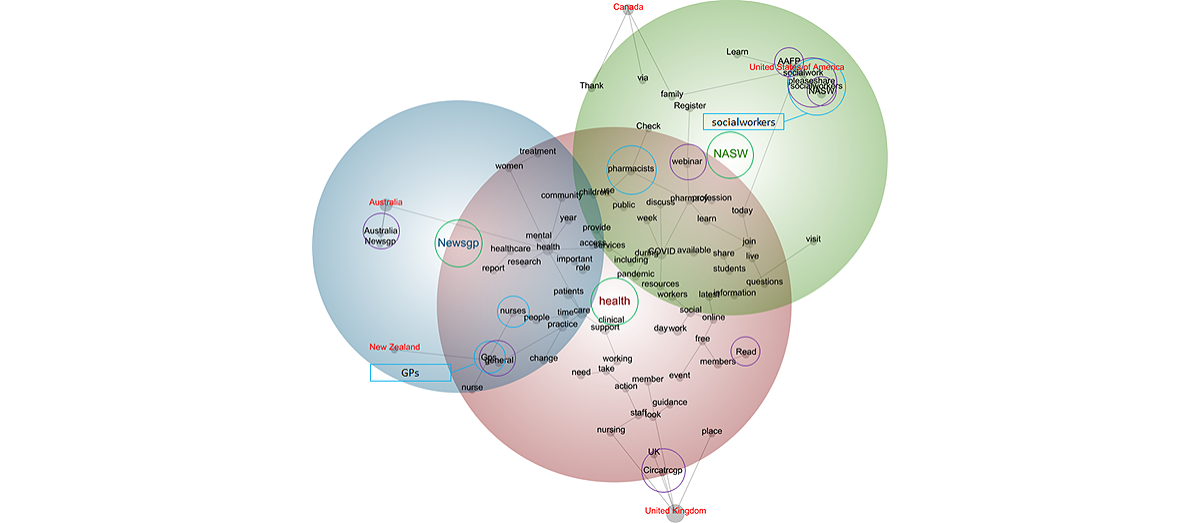
Concept map tagged by nation (visible concepts: 100%, theme size: 75%). Words circled in green denote themes, words circled in blue denote professions, and words circled in purple denote the concepts that were most likely to share content with the tag concept. In terms of themes, red denotes the most important theme, followed by green and blue. AAFP: American Association of Family Physicians; Circatrcgp: Clinical Innovation and Research Centre at the Royal College of General Practitioners; GP: general practitioner; NASW: National Association of Social Workers.

The professional associations included in this study collectively represented 5 nations: Australia, Canada, New Zealand, the United Kingdom, and the United States. The centrality of the theme *health* suggests its salience among these nations (Figure 2). However, the positions of the themes *NASW* and *Newsgp* suggest that the former had relatively greater relevance to the United States, whereas the latter had relatively greater relevance to Australia and New Zealand. This is perhaps unsurprising, given the NASW’s location and that newsGP is “the RACGP’s news hub, designed to keep Australian GPs informed about the latest in general practice” [89]. However, once again, the absence of a concept that denoted physiotherapy is noteworthy, suggesting that discourse pertaining to this profession—regardless of origin—was not prominent within the data set compared with the other 4 professions. This suggests that this pattern tended to be similar across the 5 nations.

The position of each tag concept—specifically, the 5 nations—suggests that discourse within the tweets was influenced by geography (Table 3); for instance, tweets issued by professional associations in Australia were most likely to refer to the concept *Newsgp* (100%); tweets from Canada were most likely to speak of the concept *webinar* (39%); tweets from New Zealand were most likely to allude to the concepts *general* (5%), *GPs* (5%), and *Read* (5%); tweets from the United Kingdom typically referred to the concept *Circatrcgp* (100%)—a reference to the Clinical Innovation and Research Centre at the Royal College of General Practitioners; and tweets from the United States tended to mention the concepts *AAFP*, which refers to the American Association of Family Physicians (100%), *NASW* (100%), and *pleaseshare* (100%). The close relationships between the tag concepts (as calculated using Bayesian reasoning [66]), that is, the nations, and the concepts that were specific to particular professional associations, such as *Newsgp*, *Circatrcgp*, *AAFP*, and *NASW*, are unsurprising; for instance, given that newsGP is “the RACGP’s news hub” [89], it is unsurprising that it is in close proximity to the concept tag *Australia*. However, it is noteworthy that tweets from the Canadian and New Zealander professional associations were unlikely to mention discourse specific to each of these nations or the professional associations therein; for instance, rather than tweeting about resources specific to Canadian or New Zealander professionals, they promoted more generic information via webinars or written material:

There s still time to register for today s special edition of the Coffee with Claire webinars. Topic: 2020 and beyond: the future of #Nursing and #midwifery. Join us 12 p.m. ET. Register...#yearofthenurseandmidwife

The Pharmaceutical Society of NZ agrees with...[a representative of] the Immunisation Advisory Centre, who said recently that a measles outbreak in New Zealand “should not be happening.” Read press release

**Table 3:** Top 3 likely concepts for each tag concept.

Tag concept and concept		Count (likelihood: %)
**Australia**		
	Newsgp	785 (100)
	Australia	669 (99)
	general	479 (49)
	GPs^a^	1453 (49)
**Canada**		
	webinar	491 (39)
	Check	277 (33)
	family	333 (33)
**New Zealand**		
	general	44 (5)
	GPs	136 (5)
	Read	71 (5)
**United Kingdom**		
	Circatrcgp^b^	596 (100)
	UK	780 (93)
	guidance	556 (82)
**United States**		
	AAFP^c^	611 (100)
	NASW^d^	2721 (100)
	pleaseshare	498 (100)
**General Practice**		
	Newsgp	785 (100)
	Circatrcgp	596 (100)
	AAFP	611 (100)
**Nursing**		
	nursing	1891 (98)
	nurse	695 (96)
	nurses	1213 (94)
**Pharmacy**		
	pharmacy	1305 (99)
	pharmacists	1551 (98)
	Register	295 (28)
**Physiotherapy**		
	profession	231 (21)
	treatment	92 (18)
	members	315 (15)
**Social Work**		
	NASW	2733 (100)
	socialworkers	800 (100)
	socialwork	570 (100)
	pleaseshare	498 (100)

^a^GP: general practitioner.

^b^Circatrcgp: Clinical Innovation and Research Centre at the Royal College of General Practitioners.

^c^AAFP: American Association of Family Physicians.

^d^NASW: National Association of Social Workers.

**Figure 3:**
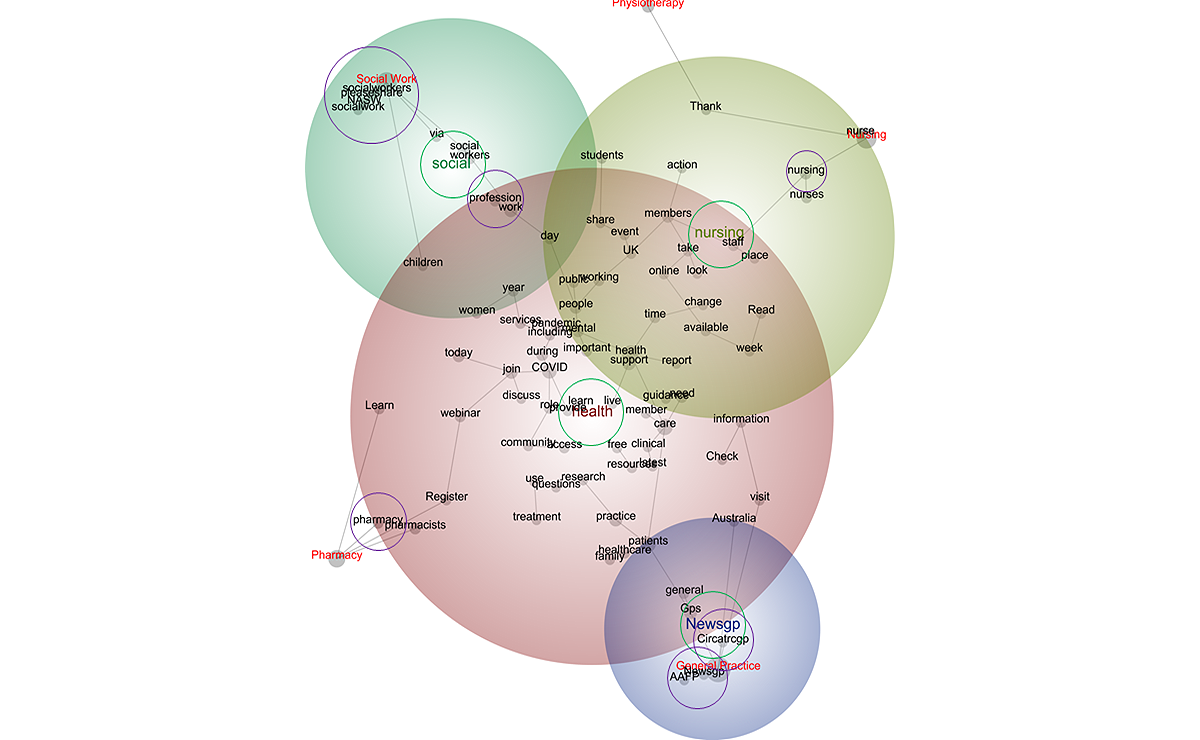
Concept map tagged by profession (visible concepts: 100%, theme size: 75%). Words circled in green denote themes, and words circled in purple denote the concepts that were most likely to share content with the tag concept. In terms of themes, red denotes the most important theme, followed by yellow, green, and purple. Circatrcgp: Clinical Innovation and Research Centre at the Royal College of General Practitioners; GP: general practitioner; NASW: National Association of Social Workers.

The associations also represented 5 professions: general practice, nursing, pharmacy, physiotherapy, and social work. The centrality of the theme *health* suggests its salience among these professions (Figure 3). However, unsurprisingly, the positions of the themes *nursing*, *social*, and *Newsgp* suggest that they had relatively greater relevance to the professions nursing, social work, and general practice, respectively.

The position of each tag concept—specifically, the 5 professions—suggests that discourse within the tweets was influenced by profession (Table 3); for instance, tweets issued by professional associations for GPs were most likely to refer to the concepts *Newsgp* (100%), *Circatrcgp* (100%), and *AAFP* (100%); tweets from professional associations for nurses were most likely to speak of the concept *nursing* (98%); tweets from professional associations for pharmacists were most likely to allude to the concept *pharmacy* (99%); tweets from professional associations for physiotherapists typically referred to the concept *profession* (21%); and tweets from professional associations for social workers tended to mention the concepts *NASW* (100%), *socialworkers* (100%), *socialwork* (100%), and *pleaseshare* (100%). The close relationships between the tag concepts (as calculated using Bayesian reasoning [66]), that is, the professions, and the concepts that were specific to particular professions or professional associations, such as *nursing* and *pharmacy*, are perhaps expected. However, it is noteworthy that tweets from physiotherapists’ professional associations were most likely to mention discourse pertaining to *profession* (although it is not the purpose of this study to clarify the reasons for this finding, it suggests a heightened need to champion this allied health care role as a profession):

Make a difference by shaping our profession. The APA is taking expressions of interest from physios to participate in two new digital projects funded by the PRF. Click below to participate

This week s CSP Newscast discusses student placements and how education has adapted...[we] then reflect...on how the profession has adapted and looks to the future. Listen now

## Discussion

### Principal Findings

Despite the increasing importance of interprofessional care for patient well-being, health care professionals do not always work together to deliver care throughout the patient journey [15,18]. The salience of professional identity raises the question of what cues professional associations provide to their members regarding their respective roles in patient care. In addition, the role of national culture in influencing managerial practices suggests that there is also a question of how the messaging of professional associations varies across nations.

To address these questions, a large corpus of tweets was examined to reveal the cues that professional associations disseminate. Specifically, the tweets of 25 professional associations were examined lexically. These associations represent 5 professions with key roles in community-based health care across 5 nations. This analysis clarified the cues that professional associations disseminate via Twitter, communicating to members and others what is (and is not) important.

From the lexical analysis, 7 key findings are noteworthy. First, the discourse within the tweets was devoid of references to interprofessional care. Irrespective of the profession represented by the associations or their geographical location, they were largely silent on interprofessional care. From a social identity perspective, this implies that professional associations are not *scripting* their members regarding the importance of interprofessional care.

Second, within the discovery mode concept map and the concept map tagged by nation, there was no concept pertaining to physiotherapists. Thus, this profession was not salient among the corpus of tweets from the 25 associations.

Third, although all the professions represented within this study support patients, the concept pertaining to GPs within the discovery mode concept map was most likely to be connected with the concept *patients*. Thus, when the tweets spoke of the other 4 professions, they were unlikely to speak of patients.

Fourth, despite the continued importance of evidence-based practice in all health care professions [90,91], within the discovery mode concept map, the tweets pertaining to pharmacists were most likely to be connected with tweets pertaining to *latest* and *research*. As such, the tweets about the other 4 professions were unlikely to speak of *latest* and *research*. This might demonstrate an effort to increase the stature of pharmacy—perhaps more of a competitive strategy than a cooperative one.

Fifth, within the discovery mode concept map, tweets about social workers were unlikely to be connected with the concepts *health* or *care* compared with tweets about the 4 other professions. Instead, tweets about social workers were relatively more likely to direct tweet recipients to particular Twitter handles or websites; although this might serve to broker connections, these connections did not explicitly speak to interprofessional cooperation, perhaps representing a missed opportunity. Nevertheless, collectively, these findings demonstrate how discourse regarding social workers differed from that regarding their professional counterparts.

Sixth, notwithstanding a few exceptions, the findings across the different nations were generally similar, suggesting their generality. With reference to the exceptions, tweets disseminated by professional associations in Canada and New Zealand encouraged recipients to access broad information that was not nation specific. Conversely, tweets disseminated by professional associations in Australia, the United Kingdom, and the United States tended to direct recipients to geographically specific information. Thus, whereas those in Canada and New Zealand facilitated connections to varied sources of information, those in Australia, the United Kingdom, and the United States drew attention to local content.

Seventh and last, the cues of the professional associations were also influenced by the profession they represented; for instance, tweets pertaining to physiotherapists were more likely to refer to discourse pertaining to *profession*, suggesting a need to raise the profile of this specialty as a profession.

### Comparison With Prior Work

The 5 nations represented in this study all emphasize rationalization, coordination, integration, a patient-centered approach, and technological change, as well as managerial governance, as espoused by new public management [92,93]. Collectively, these emphases influence the roles and competencies of health care professionals; how they enact these roles and competencies, independently and collaboratively; and the position of the health system. In this context, health care professionals use social media to voice, defend, and restore their professional identity [94].

Previous research emphasized how cultural differences can shape the nature of professional collaboration [62]. Contrary to the framework developed by Hofstede [61], this study found no evidence of national differences in professional association social media emphasis on individualism versus collectivism, power distance, uncertainty avoidance, power versus nurturing, or long-term versus short-term orientation. Despite the international importance of interprofessional care [4], discourse within the tweets from 25 professional associations revealed no explicit references to interprofessional care. Instead, the discourse largely ostracized references to physiotherapy. Furthermore, the discourse on particular professions was likely to be coupled with discourse on what they deemed to be important, which was demonstrated by the connections between *GPs* and *patients*, *pharmacists* and *latest* and *research*, and *Physiotherapy* and *profession*. These findings reveal similar patterns across the sample of nations, failing to find support for cultural differences. Given this transnational view of health care, if professional associations (are to) have a fundamental role in shaping professional identity [47,48], it is essential that their messaging recognizes the complementary roles of different professions.

### Limitations

Despite the value of the findings presented in this paper, 4 methodological limitations warrant mention. First, given the purposeful selection of professional associations in particular nations, we do not claim that the findings can be generalized to other professional associations or other nations. Second, given that the study solely involved the analysis of tweets, which are limited in content [95], professional associations might communicate different (and more detailed) messages via alternative platforms. Third, because the data set was collected during the COVID-19 pandemic, the motif and regularity of the professional associations’ tweets might have differed from the tweets they post during nonpandemic periods, thus limiting the applicability to such periods. Fourth and last, the use of Leximancer moderates the researcher’s interpretive skills, which some argue is the key to robust qualitative research [79,96].

### Conclusions

This study reveals how the professional associations of 5 health care professions, across 5 nations, used Twitter to communicate *top-of-mind* or salient themes. Specifically, the lexical analysis of a large corpus of tweets clarified what they communicated and how they communicated these messages. Furthermore, the analysis highlighted how communication compared among professions as well as among nations.

Considering extant research [4,47,48,61,62], the contributions of this study are 3-fold. First, this study demonstrated an absence of discourse regarding interprofessional care and its importance in health care across 5 professions and 5 countries; in fact, discourse regarding physiotherapists was entirely absent among the remaining professions. Given the importance of interprofessional care and its contribution to the performance of health care systems [97], this finding is important because it highlights that professional associations have not yet been able to exploit the opportunity afforded by modern communication media to raise this issue and make it salient to their members and beyond. Second, there was evidence of posturing and efforts to raise the profile of particular interests. Although this is unsurprising for membership associations, it suggests that to some extent they might foster competitive rather than cooperative stances toward other health care professions. This finding lends a degree of support to the view that “managed competition” among these professions might support the pursuit of efficiency goals at the expense of patient-centered care [98], reinforcing the previous point about the lack of discourse regarding interprofessional care. Third and last, these patterns were similar across all 5 countries, despite their differing cultures and economic systems. This finding provides an interesting point of departure from previous research examining communication between GPs and patients. Specifically, Meuweesen et al [99] found that wealth and national culture dimensions influence medical communication among various European countries. Our study provides robust evidence suggesting that national culture does not substantially contribute to differences in how professional associations communicate via Twitter.

The contributions offered by this study have clear implications for scholars and professional associations. For scholars, they open opportunities for further research on the relationship among social media, the professional identity of health care professionals, and interprofessional care. Specifically, future research is needed to clarify how social media platforms compare with other forms of collective spaces, such as communities of practice, in maintaining or altering professional identity and promoting interprofessional practices. Furthermore, given that the findings revealed a transnational view of health care across 5 nations, there is opportunity to test similar approaches to foster interprofessional care across these nations as well as extend the study further afield (eg, Asia, Africa, and South America) to determine the limits of this transnational view.

For professional associations, the findings suggest that there is much they can do to strengthen their connection with their profession as well as other health care professions; for instance, they can assert, more regularly and clearly, how the profession they represent complements other health care professions and how the professionals they represent can enact interprofessional care for the benefit of patients and carers. However, to promote interprofessional care in a sustainable way, associations will need to do this in ways that attract as well as appeal to their members. This is likely to require “attention to others” [100], that is, a consideration of the social media content their members engage with and respond to as well as an active presence on social media platforms to demonstrate responsiveness. The need to explicitly promote interprofessional care is particularly important now, given that COVID-19 has drawn heightened attention to interprofessional care [101,102]. Future efforts might test the extent and duration of this trend, as well as the potentially evolving roles of professional associations in promoting related norms.

## Abbreviations

AAFP: American Association of Family Physicians
Circatrcgp: Clinical Innovation and Research Centre at the Royal College of General Practitioners
GP: general practitioner
NASW: National Association of Social Workers

## Data Availability

The data set generated and analyzed during this study is available from the corresponding author on reasonable request.
